# Supplementary material for: Adeno‐associated virus‐vectored influenza vaccine elicits neutralizing and Fcγ receptor‐activating antibodies
Source: EMBO Mol Med. 2020 Mar 12;12(5):e10938. doi: 10.15252/emmm.201910938 (PMC7207162; doi:10.15252/emmm.201910938)
Supplement: Supplementary file 1 — Appendix [file EMMM-12-e10938-s001.pdf]

Appendix

Content:

Appendix Fig. S1: Quality control of AAV-vector preparations.

Appendix Fig. S2: Comparison of immunogenicity of i.n. or i.m. applied Cal/7/9 WIV

Appendix Table S1: Immunization groups and administered vaccine doses.

Appendix Table S2: List of exact p-values

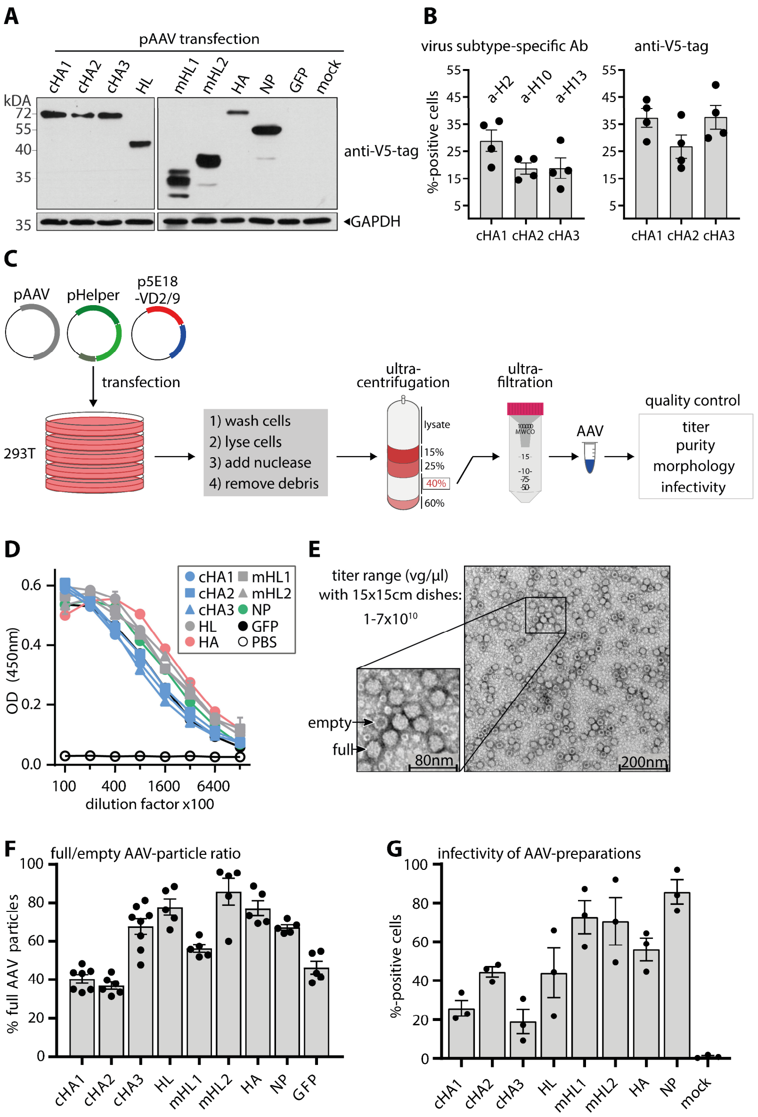

Appendix Fig. S1: Quality control of AAV-vector preparations.

A Immunoblot of 293T cell lysates obtained 48 h after transfection with AAV-vector plasmids. Antigen expression was detected with an anti-V5-tag antibody. Equal loading was controlled with a GAPDH antibody (n = 3).

B Flow cytometric analysis of 293T cells 48 h after transfection with pAAV-plasmids expressing the indicated construct. Cells were fixed, permeabilized and stained with the indicated influenza virus subtype-specific rabbit anti serum (left: α-H2, α-H10, and α-H13) or with anti-V5-tag antibody (right). Bars indicate mean ± SE (n = 4, technical duplicates).

C Schematic representation of the AAV-vector production and purification protocol

D Intact AAV9-vector capsids were detected with ADK9 antibody by ELISA (representative result, technical duplicates).

E AAV-vector preparations were quantified via quantitative real-time PCR to determine the number of encapsidated viral genomes (vg) per volume. Purity, dispersion and full-to-empty particle ratio was assessed for each AAV-batch via electron microscopy. One representative picture is shown (AAV-HA); full and empty particles are indicated by arrows.

F In a minimum of five pictures as shown in (E) full and empty AAV particles were counted and a full-to-empty ratio was calculated. Point indicate individual counts, bars indicate mean ± SE.

G Flow cytometric analysis of 293T cells 72 h after transduction with AAV-vectors at an MOI of 10^6. Cells were fixed, permeabilized and stained with anti-V5-tag antibody (n = 3). Bars indicate mean ± SE.

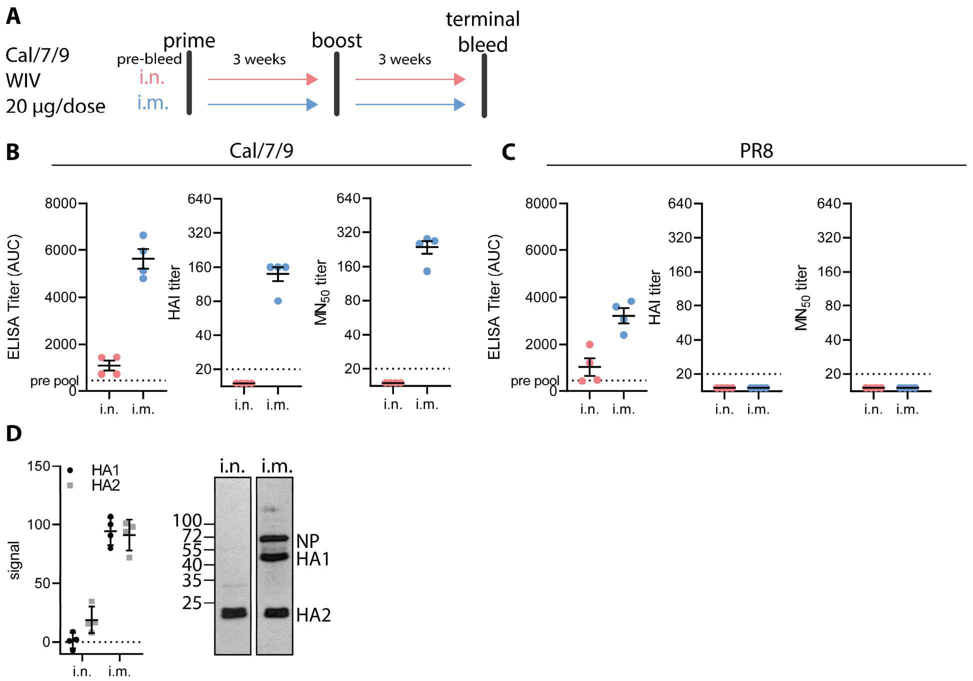

Appendix Fig. S2: Comparison of immunogenicity of i.n. or i.m. applied Cal/7/9 WIV

A Four mice per group were immunized twice in three weeks intervals with 20 µg Cal/7/9 WIV per dose in 50 µl either via the intranasal route (i.n.) our via intramuscular (i.m.) injection. Blood samples were collected before prime immunization and after the boost, in which antibody responses were analyzed.

B and C Left panel: Influenza virus-specific total IgG were measured by ELISA for each animal after the boost immunization against Cal/7/9 (B) or PR8 (C). AUC was calculated for the ELISA (points: individual animals (technical replicates), mean ± SE). Pre-serum pools were used to verify that all mice were sero-negative for influenza before the immunization. Middle panel: MN^+ antibodies were determined against Cal/7/9 (D) or PR8 (E) (points: individual animals (technical replicates), mean ± SE). Right panel: MN^+ and HAI^+ antibodies were determined against Cal/7/9 (D) or PR8 (E) (points: individual animals (technical replicates), mean ± SE).

D Immunoblot analysis of purified Cal/7/9 viruses separated under denaturing, reducing conditions. All sera were diluted 1:500 for this analysis. Intensity of HA1 and HA2 band were determined (left panel, points: indidual animals; mean ± SE). The right panel shows respective blots of one animal of the i.n. group and i.m. group, respectively.

Appendix Table S1: Immunization groups and administered vaccine doses.

Group	dose	immunization		
		#1	#2	#3
**Mouse study:**				
**AAV-HA**	10^{11} vg	AAV-HA	AAV-HA	AAV-HA
**AAV-cHA**	10^{11} vg	AAV-cHA3 (cH1/13)	AAV-cHA1 (cH1/2)	AAV-cHA2 (cH1/10)
**AAV-HL**	10^{11} vg	AAV-headless	AAV-headless	AAV-headless
**AAV-mHL1**	10^{11} vg	AAV-mHL1	AAV-mHL1	AAV-mHL1
**AAV-mHL2**	10^{11} vg	AAV-mHL2	AAV-mHL2	AAV-mHL2
**AAV-mHL1 + AAV-NP**	$5x10^{10}$ vg each	AAV-mHL1 + AAV-NP	AAV-mHL1 + AAV-NP	AAV-mHL1 + AAV-NP
**AAV-NP**	10^{11} vg	AAV-NP	AAV-NP	AAV-NP
**AAV-GFP**	10^{11} vg	AAV-GFP	AAV-GFP	AAV-GFP
**WIV**	20 µg	Cal/7/9 WIV	Cal/7/9 WIV	PBS
**Ferret study:**				
**AAV-HA**	$7.5x10^{12}$ vg	AAV-HA	AAV-HA	AAV-HA
**AAV-cHA**	$7.5x10^{12}$ vg	AAV-cHA3 (cH1/13)	AAV-cHA1 (cH1/2)	AAV-cHA2 (cH1/10)
**AAV-GFP**	$7.5x10^{12}$ vg	AAV-GFP	AAV-GFP	AAV-GFP
**QIV**	15 µg HA per component	Influsplit Tetra	Influsplit Tetra	PBS

Appendix Table S2: List of exact p-values

Figure	Panel	compared groups, p-value
Fig. 2	B	AAV-HA vs AAV-cHA <0.0001<br>AAV-HA vs AAV-NP >0.9999<br>AAV-HA vs WIV <0.0001<br>AAV-cHA vs AAV-NP 0.001<br>AAV-cHA vs WIV >0.9999<br>WIV vs AAV-NP 0.0004<br>AAV-HA vs pre <0.0001<br>AAV-cHA vs pre <0.0001<br>AAV-NP vs pre 0.001<br>WIV vs pre <0.0001
	C	AAV-HA vs AAV-cHA 0.0111<br>AAV-HA vs AAV-WIV >0.9999<br>AAV-HA vs AAV-NP >0.9999<br>AAV-HA vs AAV-GFP >0.0001<br>AAV-cHA vs WIV 0.588<br>AAV-cHA vs AAV-NP 0.0028<br>AAV-cHA vs AAV-GFP 0.7241<br>WIV vs AAV-NP 0.8128<br>WIVvs AAV-GFP 0.0023<br>AAV-NP vs AAV-GFP >0.0001
Fig. 3	C	Cal/7/9 pH 7.2 vs 5.8 >0.9999<br>Cal/7/9 pH 7.2 vs 5.4 0.8389<br>Cal/7/9 pH 7.2 vs 5.0 0.1927<br>Cal/7/9 pH 7.2 vs 4.4 0.0064<br>Cal/7/9 pH 7.2 vs DTT 0.0140<br>PR8 pH 7.2 vs 5.8 >0.9999<br>PR8 pH 7.2 vs 5.4 >0.9999<br>PR8 pH 7.2 vs 5.0 0.232<br>PR8 pH 7.2 vs 4.4 0.029<br>PR8 pH 7.2 vs DTT 0.023
	D	Cal/7/9 pH 7.2 vs 5.8 >0.9999<br>Cal/7/9 pH 7.2 vs 5.4 >0.9999<br>Cal/7/9 pH 7.2 vs 5.0 >0.9999<br>Cal/7/9 pH 7.2 vs 4.4 0.329<br>Cal/7/9 pH 7.2 vs DTT 0.029<br>PR8 pH 7.2 vs 5.8 >0.9999<br>PR8 pH 7.2 vs 5.4 >0.9999<br>PR8 pH 7.2 vs 5.0 >0.9999<br>PR8 pH 7.2 vs 4.4 >0.9999<br>PR8 pH 7.2 vs DTT >0.9999
	E	Cal/7/9 pH 7.2 vs 5.8 >0.9999<br>Cal/7/9 pH 7.2 vs 5.4 0.8389<br>Cal/7/9 pH 7.2 vs 5.0 0.1076<br>Cal/7/9 pH 7.2 vs 4.4 0.0572<br>Cal/7/9 pH 7.2 vs DTT 0.0028<br>PR8 pH 7.2 vs 5.8 >0.9999<br>PR8 pH 7.2 vs 5.4 >0.9999<br>PR8 pH 7.2 vs 5.0 >0.9999<br>PR8 pH 7.2 vs 4.4 0.73

		PR8 pH 7.2 vs DTT 0.19
	F	Cal/7/9 pH 7.2 vs 5.8 >0.9999<br>Cal/7/9 pH 7.2 vs 5.4 0.277<br>Cal/7/9 pH 7.2 vs 5.0 0.277<br>Cal/7/9 pH 7.2 vs 4.4 0.23<br>Cal/7/9 pH 7.2 vs DTT 0.057<br>PR8 pH 7.2 vs 5.8 0.54<br>PR8 pH 7.2 vs 5.4 >0.9999<br>PR8 pH 7.2 vs 5.0 >0.9999<br>PR8 pH 7.2 vs 4.4 >0.9999<br>PR8 pH 7.2 vs DTT 0.28
Fig. 5	A	AAV-HA vs AAV-GFP 0.0082<br>AAV-cHA vs AAV-GFP 0.0074<br>AAV-NP vs AAV-GFP 0.0016<br>WIV vs AAV-GFP 0.0047
	C	AAV-HA vs AAV-GFP 0.0016<br>AAV-cHA vs AAV-GFP 0.0016<br>AAV-NP vs AAV-GFP 0.0016<br>WIV vs AAV-GFP 0.0016
	E	AAV-HA vs AAV-GFP 0.0014<br>AAV-cHA vs AAV-GFP 0.0014<br>WIV vs AAV-GFP 0.3299
Fig. 6	B	AAV-HA pre vs immu #1 0.7605<br>AAV-HA pre vs immu #2 0.279<br>AAV-HA pre vs immu #3 0.0044<br>AAV-cHA pre vs immu #1 0.2098<br>AAV-cHA pre vs immu #2 0.0247<br>AAV-cHA pre vs immu #3 0.0806<br>AAV-GFP pre vs immu #1 >0.9999<br>AAV-GFP pre vs immu #2 >0.9999<br>AAV-GFP pre vs immu #3 >0.9999<br>QIV pre vs immu #1 >0.9999<br>QIV pre vs immu #2 >0.9999<br>QIV pre vs immu #3 0.2822
	C	AAV- HA d0 vs d1 >0.9999<br>AAV- HA d0 vs d2 0.256<br>AAV- HA d0 vs d3 0.3417<br>AAV- cHA d0 vs d1 >0.9999<br>AAV- cHA d0 vs d2 0.0643<br>AAV- cHA d0 vs d3 0.2319<br>AAV-GFP d0 vs d1 >0.9999<br>AAV-GFP d0 vs d2 0.0218<br>AAV-GFP d0 vs d3 0.01<br>QIV d0 vs d1 >0.9999<br>QIV d0 vs d2 0.051<br>QIV d0 vs d3 0.0192
	D	AAV- HA d0 vs d1 >0.9999<br>AAV- HA d0 vs d2 0.005<br>AAV- HA d0 vs d3 0.8965<br>AAV- cHA d0 vs d1 >0.9999<br>AAV- cHA d0 vs d2 0.005<br>AAV- cHA d0 vs d3 0.3753

		AAV-GFP d0 vs d1 0.8262<br>AAV-GFP d0 vs d2 0.0114<br>AAV-GFP d0 vs d3 0.1711<br>QIV d0 vs d1 >0.9999<br>QIV d0 vs d2 0.0279<br>QIV d0 vs d3 0.0192
	E	AAV- HA d0 vs d1 >0.9999<br>AAV- HA d0 vs d2 >0.9999<br>AAV- HA d0 vs d3 0.0643<br>AAV- cHA d0 vs d1 >0.9999<br>AAV- cHA d0 vs d2 0.6992<br>AAV- cHA d0 vs d3 0.0901<br>AAV-GFP d0 vs d1 >0.9999<br>AAV-GFP d0 vs d2 >0.9999<br>AAV-GFP d0 vs d3 0.9715<br>QIV d0 vs d1 >0.9999<br>QIV d0 vs d2 0.2319<br>QIV d0 vs d3 0.0114
	F	NT:<br>AAV-GFP vs AAV-HA 0.6204<br>AAV-GFP vs AAV-cHA >0.9999<br>AAV-GFP vs QIV 0-8955<br><br>lungs:<br>AAV-GFP vs AAV-HA 0.1025<br>AAV-GFP vs AAV-cHA >0.9999<br>AAV-GFP vs QIV >0.9999<br><br>trachea:<br>AAV-GFP vs AAV-HA 0.0224<br>AAV-GFP vs AAV-cHA >0.9999<br>AAV-GFP vs QIV >0.9999

Fig. EV1	I	AAV-HA vs AAV-cHA >0.9999<br>AAV-HA vs WIV 0.3007<br>AAV-HA vs AAV-NP 0.0001<br>AAV-HA vs AAV-GFP 0.9359<br>AAV-cHA vs WIV >0.9999<br>AAV-cHA vs AAV-NP 0.0063<br>AAV-cHA vs AAV-GFP 0.0809<br>WIV vs AAV-NP 0.2626<br>WIV vs AAV-GFP 0.0012<br>AAV-NP vs AAV-GFP <0.0001
Fig. EV2	D	only p-values for peptide of interest:<br>Pep16 0.0171<br>Pep30 0.0331<br>Pep39 0.049<br>Pep41 0.0209<br>Pep48 0.0263
	E	only p-values for peptide of interest:

		Pep44 0.128
	G	Cal/7/9 pH 7.2 vs 5.8 >0.9999<br>Cal/7/9 pH 7.2 vs 5.4 >0.9999<br>Cal/7/9 pH 7.2 vs 5.0 >0.9999<br>Cal/7/9 pH 7.2 vs 4.4 >0.9999<br>Cal/7/9 pH 7.2 vs DTT >0.9999<br>PR8 pH 7.2 vs 5.8 0.39<br>PR8 pH 7.2 vs 5.4 >0.9999<br>PR8 pH 7.2 vs 5.0 0.33<br>PR8 pH 7.2 vs 4.4 0.54<br>PR8 pH 7.2 vs DTT >0.9999
Fig. EV4	A	AAV-GFP vs AAV-HA 0.0006<br>AAV-GFP vs AAV-cHA 0.0482<br>AAV-GFP vs WIV 0.1219<br>AAV-GFP vs AAV-NP 0.2112
	B	AAV-GFP vs AAV-HA 0.001<br>AAV-GFP vs AAV-cHA 0.0095<br>AAV-GFP vs WIV 0.001<br>AAV-GFP vs AAV-NP 0.001<br>AAV-HA vs AAV-cHA >0.9999
	C	AAV-GFP vs AAV-HA 0.0064<br>AAV-GFP vs AAV-cHA 0.0152<br>AAV-GFP vs WIV 0.4358<br>AAV-GFP vs AAV-NP 0.0146
	D	AAV-GFP vs AAV-HA <0.0001<br>AAV-GFP vs AAV-cHA <0.0001<br>AAV-GFP vs WIV <0.0001<br>AAV-GFP vs AAV-NP <0.0001
	E	AAV-GFP vs AAV-HA 0.31<br>AAV-GFP vs AAV-cHA 0.4172<br>AAV-GFP vs WIV >0.9999
	F	d3:<br>AAV-GFP vs AAV-HA >0.9999<br>AAV-GFP vs AAV-cHA >0.9999<br>AAV-GFP vs WIV >0.9999<br><br>endpoint/day14:<br>AAV-GFP vs AAV-HA 0.0104<br>AAV-GFP vs AAV-cHA 0.0045<br>AAV-GFP vs WIV >0.9999
Fig. EV5	C	AAV-HA pre vs immu #1 >0.9999<br>AAV-HA pre vs immu #2 >0.9999<br>AAV-HA pre vs immu #3 >0.9999<br>AAV-cHA pre vs immu #1 >0.9999<br>AAV-cHA pre vs immu #2 >0.9999<br>AAV-cHA pre vs immu #3 0.6421<br>AAV-GFP pre vs immu #1 >0.9999<br>AAV-GFP pre vs immu #2 >0.9999<br>AAV-GFP pre vs immu #3 0.0279<br>QIV pre vs immu #1 >0.9999<br>QIV pre vs immu #2 >0.9999<br>QIV pre vs immu #3 >0.9999

	F	AAV-GFP vs AAV-HA 0.1883<br>AAV-GFP vs AAV-cHA 0.8122<br>AAV-GFP vs QIV >0.9999
	G	AAV-GFP vs AAV-HA 0.0089<br>AAV-GFP vs AAV-cHA >0.9999<br>AAV-GFP vs QIV 0.0569
	H	AAV-GFP vs AAV-HA 0.0974<br>AAV-GFP vs AAV-cHA >0.9999<br>AAV-GFP vs QIV 0.221
	I	AAV-GFP vs AAV-HA >0.9999<br>AAV-GFP vs AAV-cHA 0.2653<br>AAV-GFP vs QIV 0.6345
	J	AAV-GFP vs AAV-HA 0.5094<br>AAV-GFP vs AAV-cHA >0.9999<br>AAV-GFP vs QIV 0.4381
