# Supplementary material for: Adeno‐associated virus‐vectored influenza vaccine elicits neutralizing and Fcγ receptor‐activating antibodies
Source: EMBO Mol Med. 2020 Mar 12;12(5):e10938. doi: 10.15252/emmm.201910938 (PMC7207162; doi:10.15252/emmm.201910938)
Supplement: Supplementary file 2 — Expanded View Figures PDF [file EMMM-12-e10938-s002.pdf]

Expanded View Figures

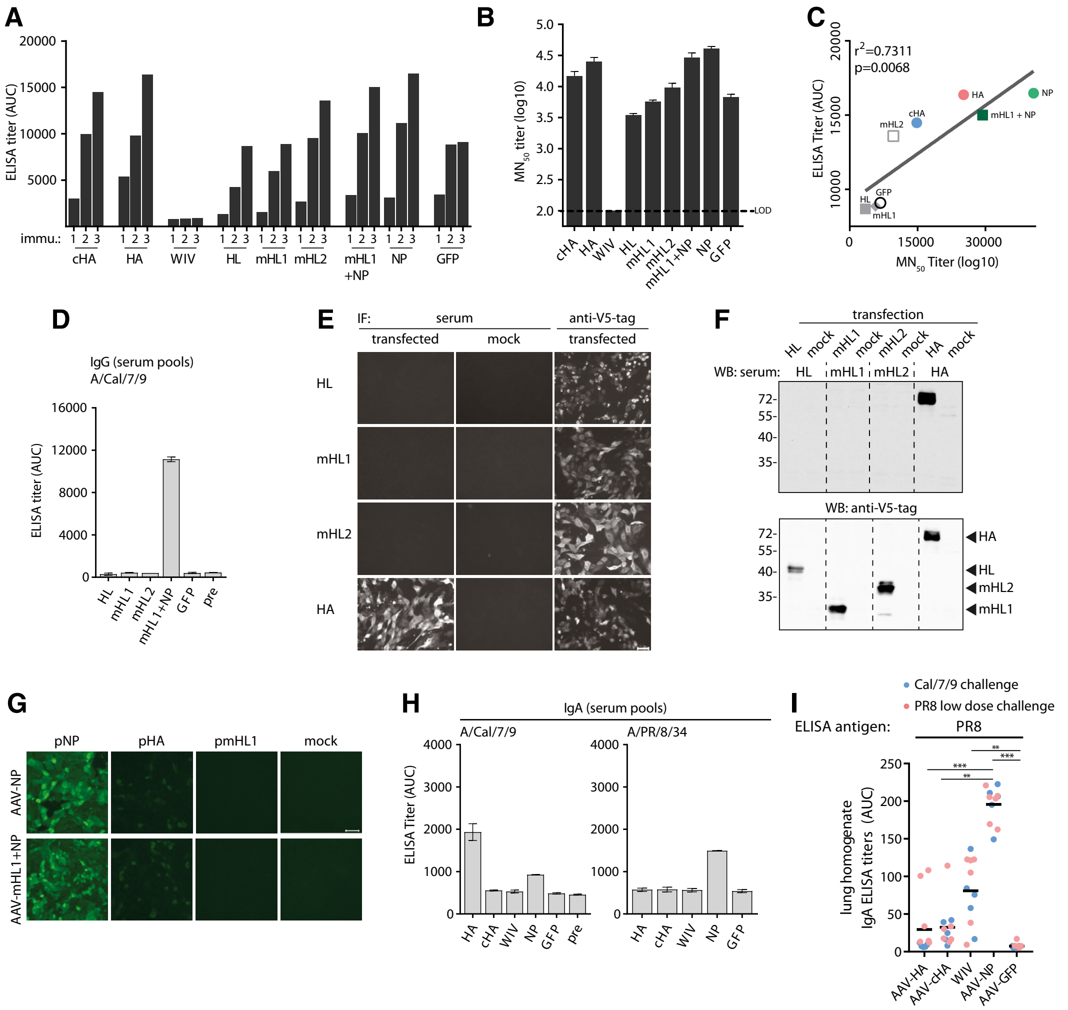

Figure EV1. Serum antibody responses against the AAV vector capsid and PR8-specific IgA.

A Total IgG ELISA titer induction over vaccination period against AAV9-vector capsids in pooled sera (AAV-HA, -cHA, -GFP, WIV $n = 18$; AAV-NP $n = 11$; AAV-HL, -mHL1, -mHL2 $n = 7$ mice per group) at indicated time points.

B AAV9-vector-neutralizing antibody titers in pooled pre-challenge sera (AAV-HA, -cHA, -GFP, WIV $n = 18$; AAV-NP $n = 11$; AAV-HL, -mHL1, -mHL2 $n = 7$ mice per group, technical duplicates). Dotted line indicates the limit of detection (LOD) at a dilution of 1:100. Mean ± SD.

C Regression analysis of total AAV9-vector IgG ELISA titers and MN_{50} titers. Coefficient of correlation (r^2) and P-value are shown.

D Total IgG ELISA titers expressed as AUC against homologous Cal/7/9 virus in pre-challenge serum pools (AAV-HA, -cHA, -GFP, WIV $n = 18$; AAV-NP $n = 11$; AAV-HL, -mHL1, -mHL2 $n = 7$ mice per group, technical duplicates) of the indicated vaccine groups. Mean ± SD.

E Immunofluorescence microscopy staining of MDCKII cells 48 h after transfection with pAAV plasmids expressing the indicated constructs. Cells were fixed, permeabilized, and stained with the indicated mouse pre-challenge serum pools or with anti-V5-tag antibody groups ($n = 2$, technical duplicates) (bar, 50 μm).

F Immunoblot analysis with lysates obtained from 293T 48 h after transfection with pAAV plasmids expressing the indicated constructs. Immunoblot was performed with the indicated pre-challenge mouse serum pools (top, AAV-HA $n = 18$, AAV-HL, -mHL1, -mHL2 $n = 7$ mice per group). Hereafter, membranes were stripped and stained with anti-V5-tag antibody (bottom). Position of detected protein bands is indicated to the right (bottom).

G Immunofluorescence microscopy staining of MCDKII cells 24 h after transfection with wild-type Cal/7/9 NP or HA and mHL1 plasmids with pre-challenge serum from AAV-NP or AAV-mHL1 + NP immunization groups ($n = 2$, technical duplicates) (bar, 50 μm).

H IgA ELISA titers against Cal/7/9 or PR8 virus in pooled pre-challenge sera (AAV-HA, -cHA, -GFP, WIV $n = 18$; AAV-NP $n = 11$ mice per group, technical duplicates). Mean ± SD.

I IgA ELISA titers in post-challenge lung homogenates of individual mice of the Cal/7/9 and PR8 low-dose challenge groups of the indicated vaccine groups against PR8. Statistical significance between vaccine groups was determined using Kruskal–Wallis test with Dunn's multiple comparison testing (**$P < 0.01$, ***$P < 0.001$). Lines indicate mean. ELISAs were done in technical duplicates.

Source data are available online for this figure.

© 2020 The Authors

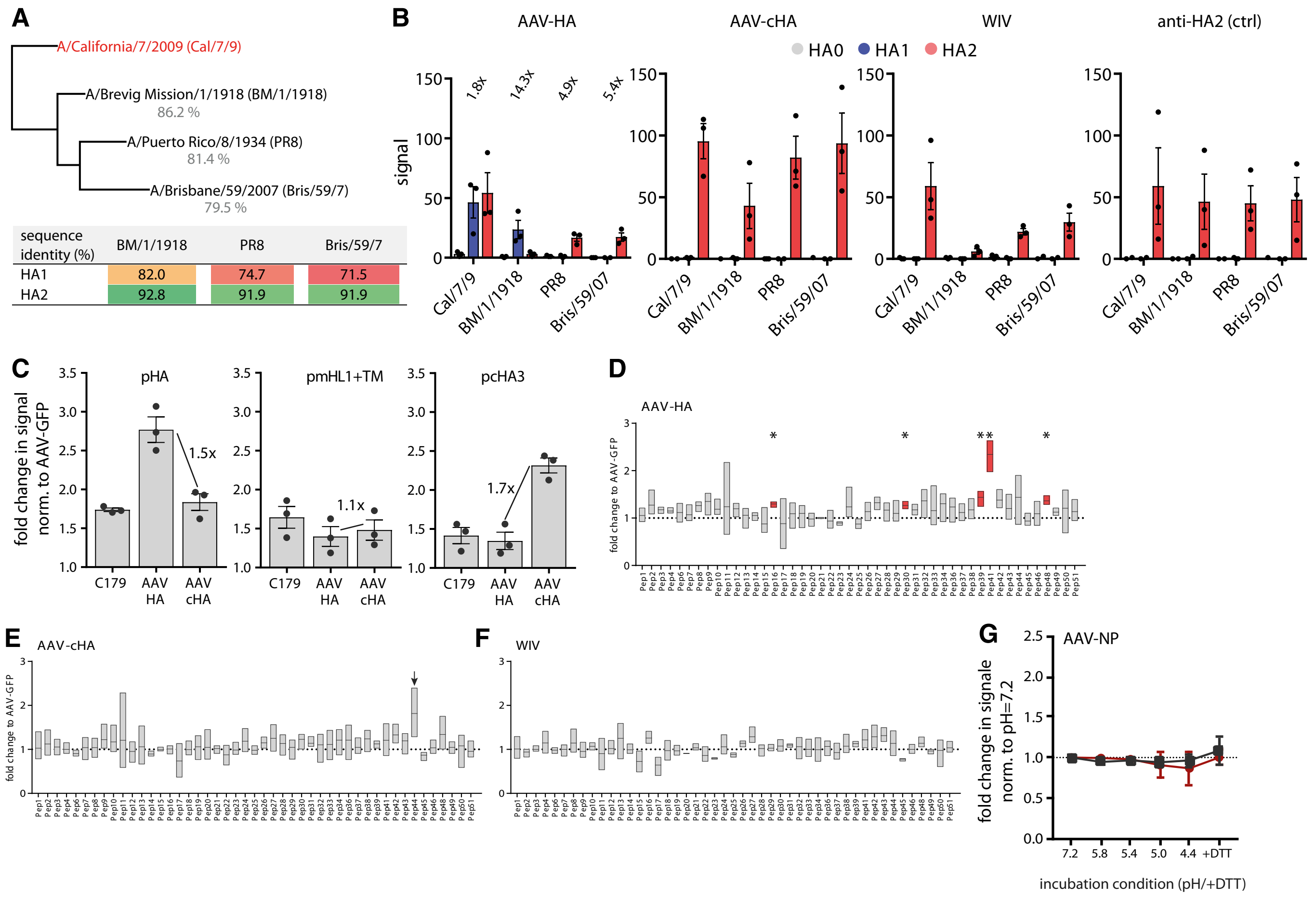

Figure EV2. Induction of distinct antibody profiles after vaccination with AAV-vectored vaccines or WIV.

A Phylogenetic tree of the complete HA sequences of the four H1N1 viruses used for immunoblot analysis with mouse serum. The table shows the amino acid identities of the H1N1 viruses HA1 or HA2 subdomain compared to Cal/7/9 HA1 and HA2, respectively, as determined with Geneious 11.1.5 software.

B Quantification of immunoblot data as shown in Fig 3A. Intensities of bands were analyzed using ImageJ. Dots indicate individual experiments, bars mean ± SE (n = 3). Numbers in the AAV-HA panel indicate fold-change of signal of the HA2 band of the AAV-HA compared to the AAV-cHA group.

C In-cell ELISA with transfected MDCKII cells expressing either Cal/7/9 full-length HA (pAAV-HA), the HA-stalk (pAAV-mHL1 + transmembrane region), or chimeric HA consisting of the Cal/7/9 HA stalk and the H13 HA head (pcHA3). Detection of the HA constructs was done with indicated mouse serum at a 1:250 dilution or with the conformational stalk antibody C179. Dots indicate individual experiments, bars mean ± SE (n = 3, technical triplicates). Numbers indicated the fold-change of signal between AAV-HA and AAV-cHA groups tested against the indicated HA construct.

D Results of 15-mer Cal/7/9 peptide screen with AAV-HA pooled pre-challenge serum (n = 18 mice). Peptides were coated in 96-well plates, incubated with serum before binding was detected with a HRP-coupled secondary antibody. Data are shown as fold induction over AAV-GFP signal intensity (n = 3, technical duplicates). Statistical significance between each peptide signal and the baseline value 1 (dotted line) was determined using one-sample t-test (*P < 0.05). Floating bars represent mean ± range.

E, F Results of epitope screen with 15-mer peptides with pooled AAV-cHA and WIV sera (n = 18 mice per group). For AAV-cHA, peptide #44, showing increased but non-significant binding, is indicated by an arrow. Statistical significance between each peptide signal and the baseline value 1 (dotted line) was determined using one-sample t-test. Floating bars represent mean ± range (n = 3, technical duplicates).

G Binding of AAV-NP pre-challenge sera (n = 11 mice) to Cal/7/9 or PR8 virus after incubation of the virions at pH = 7.2, 5.8, 5.4, 5.0, 4.4 or 4.4 + DTT. Bars represent mean ± SD (n = 3).

 © 2020 The Authors

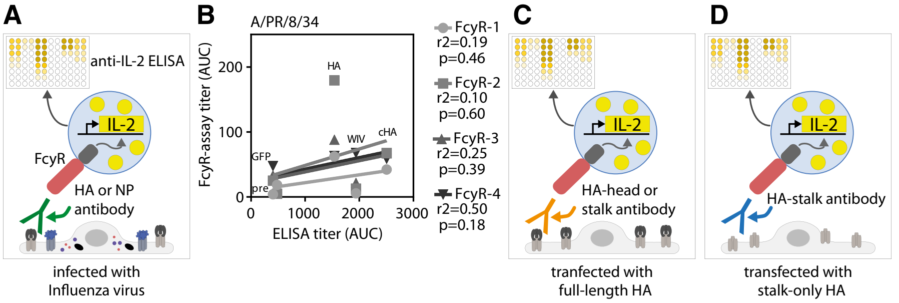

Figure EV3. Principle of the FcγR assay and correlation between PR8 FcγR titer and total IgG ELISA titer.

A Scheme of FcγR assay setups used to detect FcγR-activating antibodies against all viral proteins. MDCKII cells were either infected with influenza virus before pre-challenge sera and FcγR-reporter cells were added. Upon activation of the FcγR, IL-2 is produced within the FcγR-reporter cell, which is quantified by anti-IL-2 ELISA.

B Correlation between total influenza IgG ELISA titers and FcγR-activating antibody titers against PR8 virus. Coefficient of correlation (r^2) and *P*-value is shown for each receptor.

C, D Scheme of FcγR assay setups used to detect FcγR-activating antibodies against the complete HA protein (C) or the HA-stalk domain (D). Uninfected MDCKII cells were transfected with wild-type HA (pAAV-HA) or a stalk-only construct (pAAV-mHL1 + transmembrane region) before pre-challenge sera and FcγR-reporter cells were added. Upon activation of the FcγR, IL-2 is produced within the FcγR-reporter cell, which is quantified by anti-IL-2 ELISA.

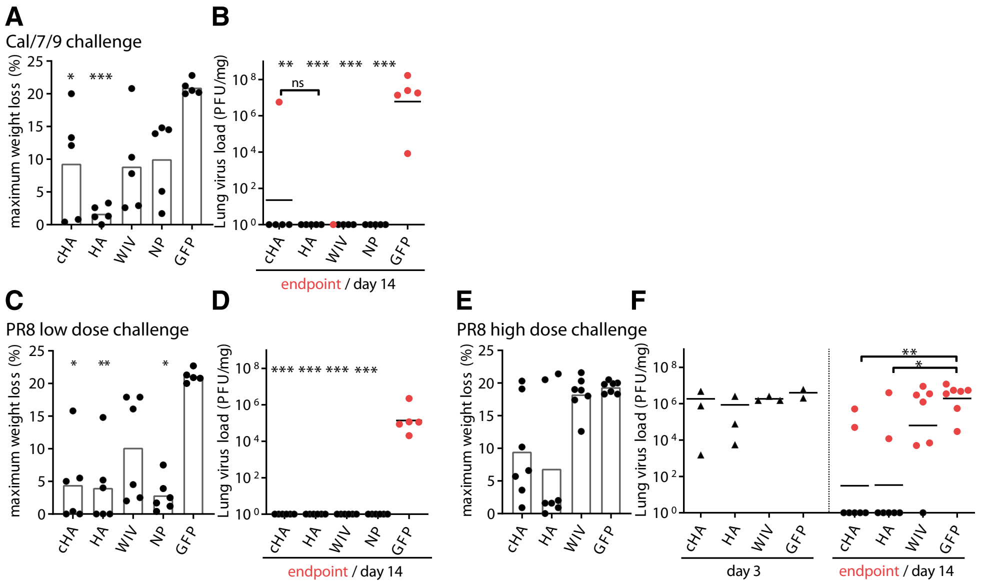

Figure EV4. Weight loss and virus load in mouse lungs and survival of AAV- headless HA-immunized mice.

A, B Maximum weight loss during challenge period, and lung virus load at the individual endpoint (red points) or 14 days post-infection (black points) with Cal/7/9 ($n = 5$ mice per group).

C, D Maximum weight loss during challenge period (C), and lung virus load at the individual endpoint (red points) or day 14 post-infection (black points) (D) with the lower dose of PR8 9 ($n = 6$ mice per group).

E, F Maximum weight loss during challenge period (E, $n = 7$ mice per group), and lung virus load at day 3 post-infection (black triangles) or at the individual endpoint (red points) or 14 days post-infection (black points) (F, day 3 $n = 3$ mice per group, endpoint/day 14 $n = 7$ mice per group) with the higher dose of PR8.

Data information: (A, C, E) Bars indicate mean. (B, D, F) Lines indicate mean). (A–F) Statistical significance between the negative control (AAV-GFP) and vaccine groups was determined using Kruskal–Wallis test with Dunn's multiple comparison testing (*$P < 0.05$; **$P < 0.01$; ***$P < 0.001$).

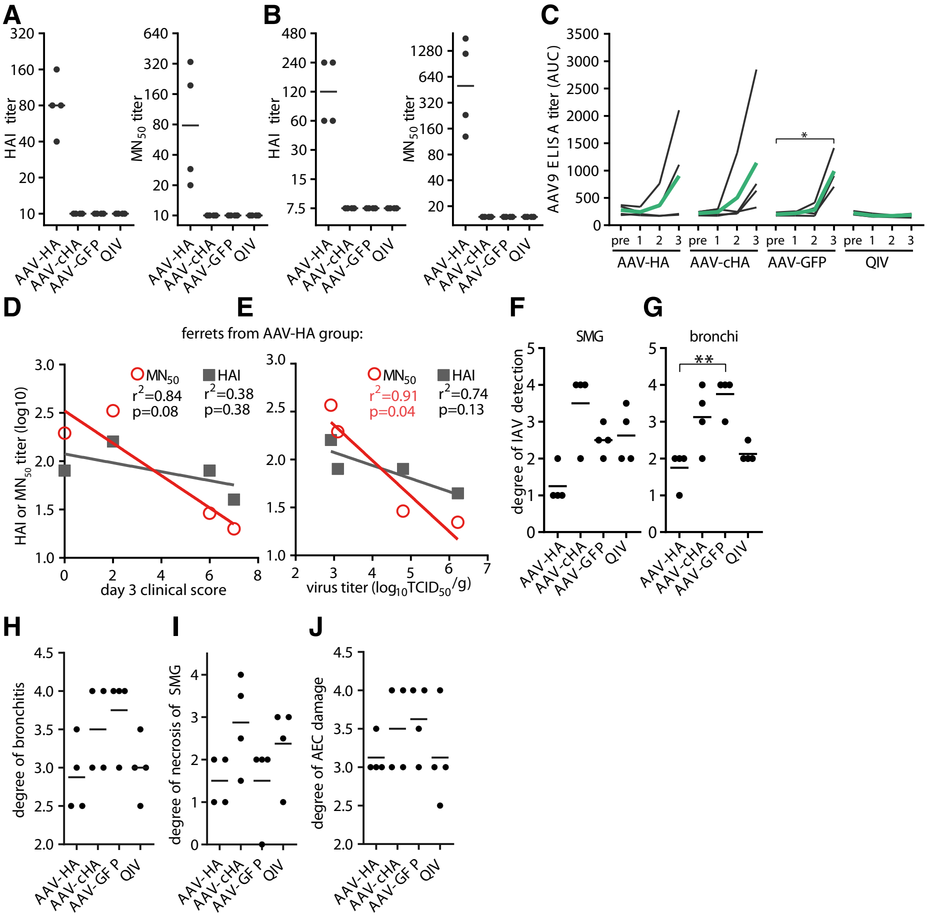

Figure EV5. Influenza MN, HAI, and AAV9 serum antibody titers in ferrets.

A HAI and MN_{50} titers in individual ferret sera against Cal/7/9. Assay done in technical duplicates.

B HAI and MN_{50} titers of individual ferret sera against Michigan/45/2015 (H1N1)pdm. Assay done in technical duplicates.

C AAV9-specific serum antibody titers in individual ferret sera at indicated time points. Black lines: individual animals, green lines: mean titer. Statistical significance between pre-serum and immune serum 1, 2, or 3 was determined using Friedman test with Dunn's multiple comparison testing (*$P < 0.05$). Assay done in technical duplicates.

D Association between clinical score at 3 dpi and MN_{50} or HAI antibody titers of ferret of the AAV-HA group.

E Association between virus titer in nasal turbinates and MN_{50} or HAI antibody titers of ferrets of the AAV-HA group. Coefficient of correlation (r^2) and P-value for correlations is shown. Symbols represent individual animals and the lines the linear regression curve.

F, G Scoring of IHC staining of influenza A virus antigen expression in submucosal glands (SMG) or bronchi of ferrets. Lines indicate mean. Statistical significance between AAV-GFP group and other groups determined using Kruskal–Wallis test with Dunn's multiple comparison testing (**$P < 0.01$).

H–J Scoring of histo-pathologic assessment of fixed lung tissue. Degree of bronchiolitis, necrosis of submucosal glands (SMG). and alveolar epithelial cells (AEC) were examined by board-certified pathologists.

Data information: Dots: individual animals; bar: geometric mean.

© 2020 The Authors
