# Supplementary figures and images for: Adeno‐associated virus‐vectored influenza vaccine elicits neutralizing and Fcγ receptor‐activating antibodies
Source: EMBO Mol Med. 2020 Mar 12;12(5):e10938. doi: 10.15252/emmm.201910938 (PMC7207162; doi:10.15252/emmm.201910938)

mouse-anti-V5-tag antibody

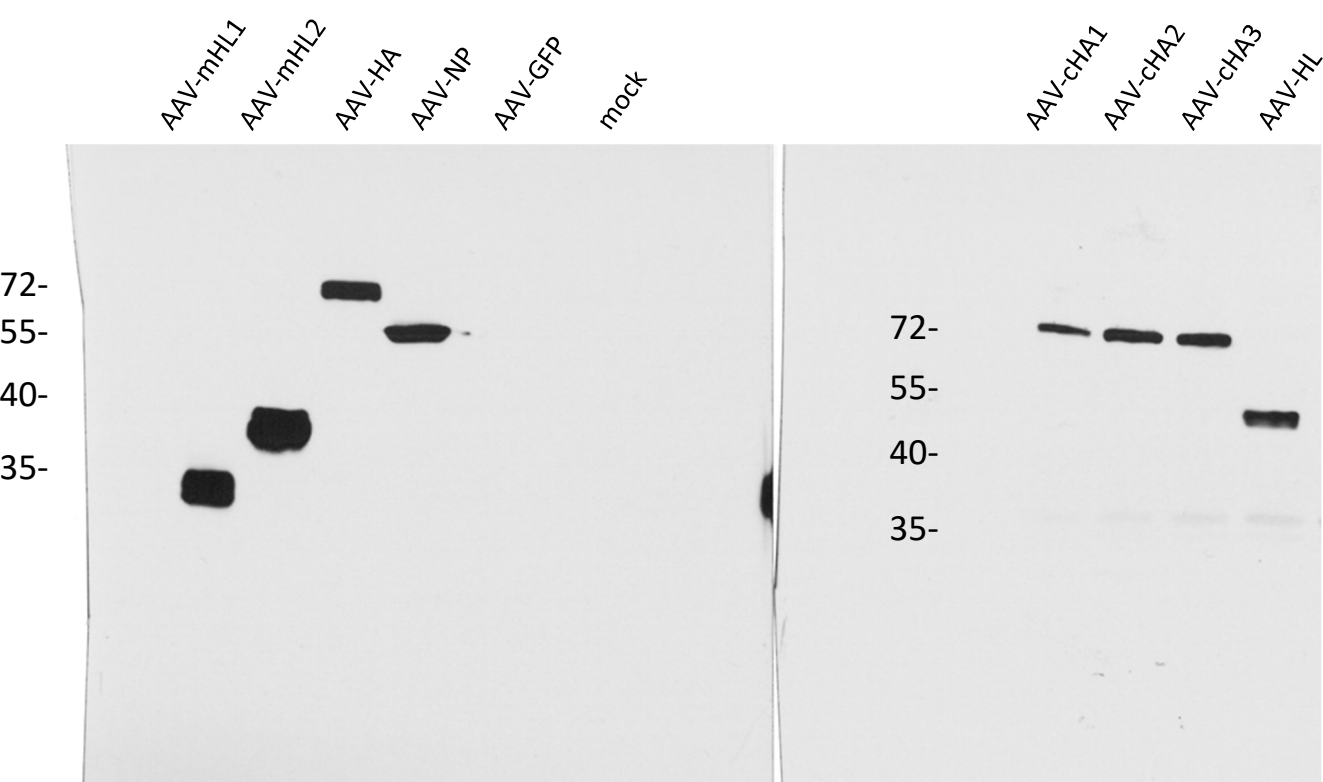

rabbit-anti-GAPDH antibody (after Stripping of the above blot)

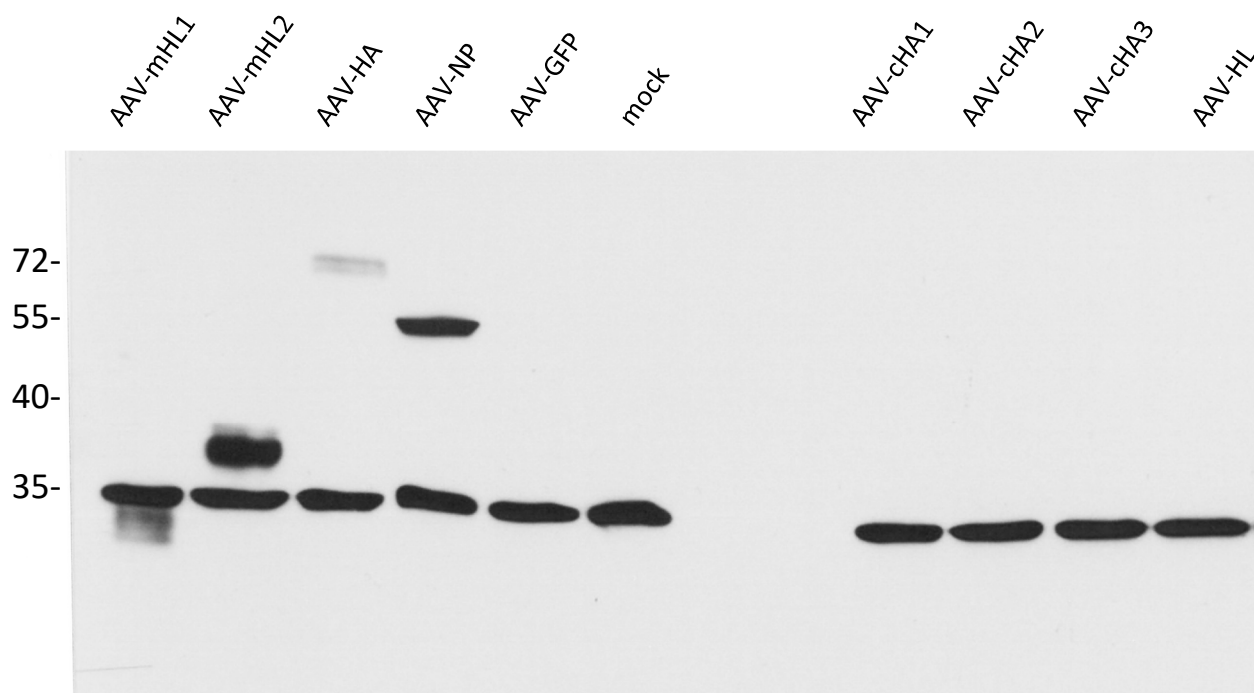

Supplement: Supplementary file 3 — Source Data for Figure 1 [file EMMM-12-e10938-s003.pdf]

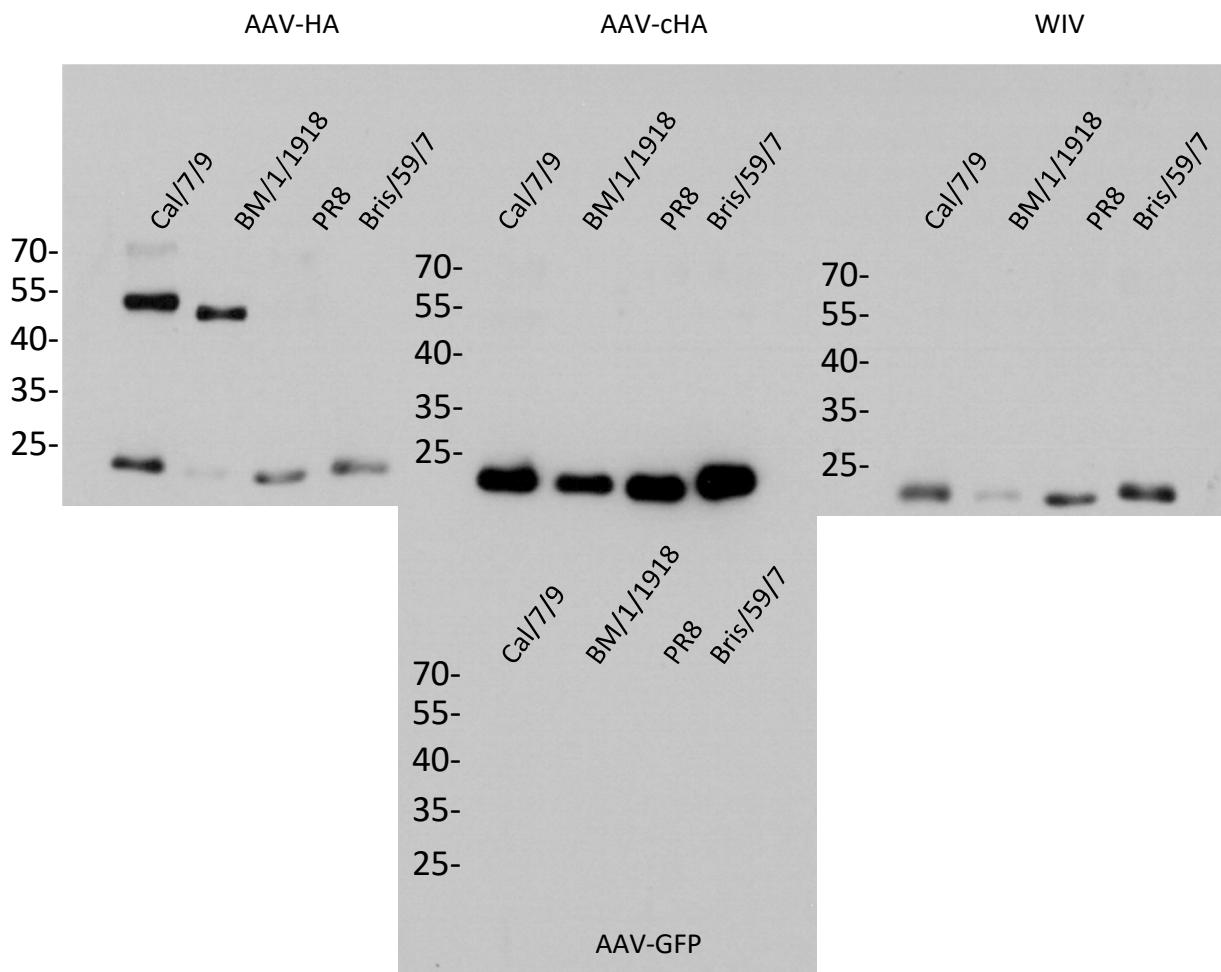

anti-HA2 (ctrl)

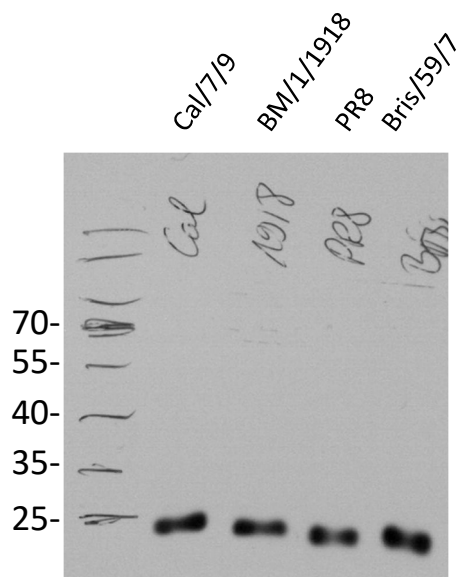

Supplement: Supplementary file 4 — Source Data for Figure 3 [file EMMM-12-e10938-s004.pdf]

mouse-anti-V5-tag antibody

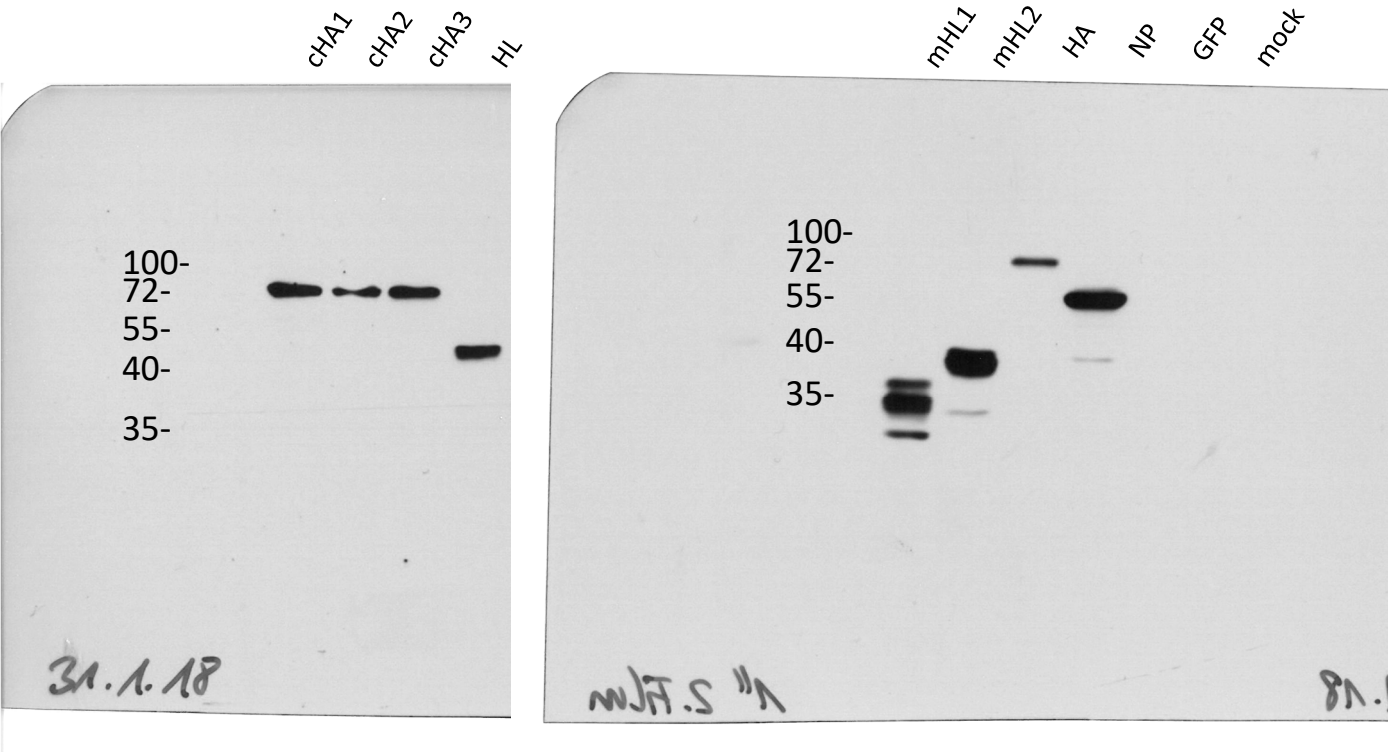

rabbit-anti-GAPDH antibody (after Stripping of the above blot)

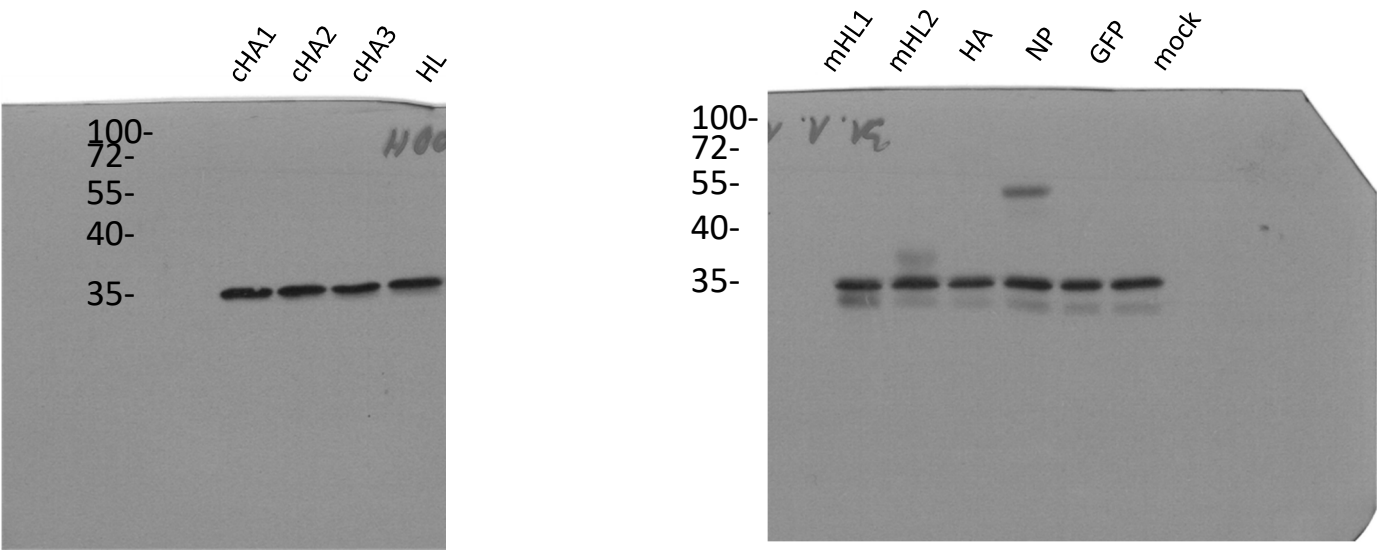

Supplement: Supplementary file 5 — Source Data for Expanded View [file EMMM-12-e10938-s005.zip › emmm201910938-sup-0003-SDataAppendixFigS1A.pdf]

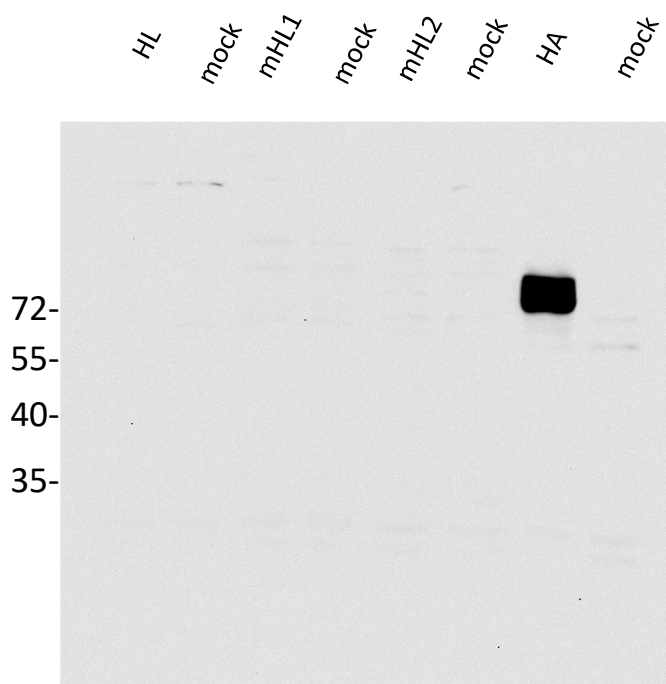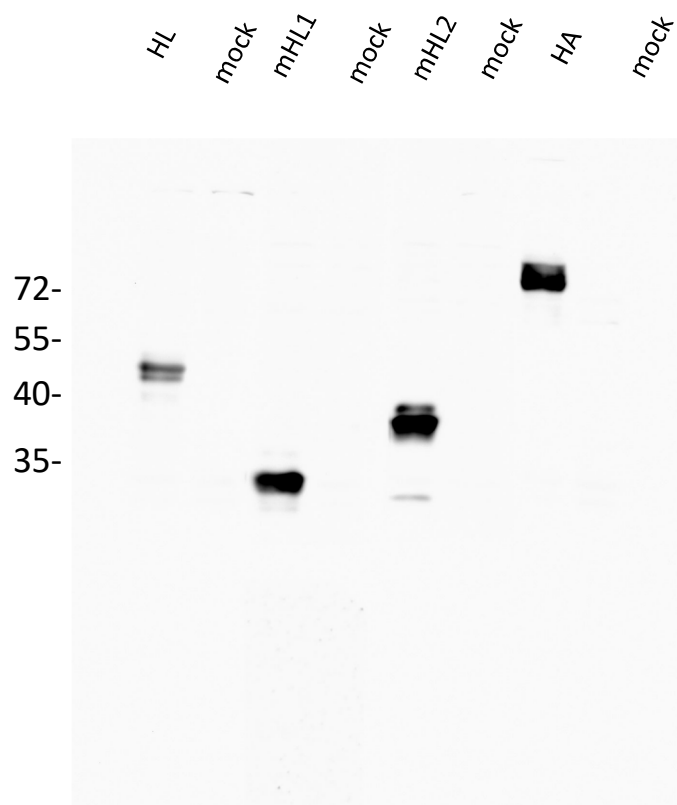

Supplement: Supplementary file 5 — Source Data for Expanded View [file EMMM-12-e10938-s005.zip › emmm201910938-sup-0006-SDataFigEV1F.pdf]
